# Comparison of Easy-to-Use Bronchiolitis Scores in the Post-COVID-19 Era—An Observational Study

**DOI:** 10.3390/children10121834

**Published:** 2023-11-21

**Authors:** María del Mar Martín-Latorre, Leticia Martínez-Campos, Manuel Martín-González, Gracia Castro-Luna, David Lozano-Paniagua, Bruno José Nievas-Soriano

**Affiliations:** 1 Department of Pediatrics, Torrecárdenas University Hospital, 04009 Almeria, Spain; mariam.martin.latorre.sspa@juntadeandalucia.es (M.d.M.M.-L.); leticia.martinez.sspa@juntadeandalucia.es (L.M.-C.); mmg189@ual.es (M.M.-G.); graciacl@ual.es (G.C.-L.); 2 Nursing, Physiotherapy, and Medicine Department, University of Almeria, 04120 Almeria, Spain; brunonievas@ual.es

**Keywords:** bronchiolitis, severity, score, SARS-CoV-2, Tal score

## Abstract

In the post-restrictions COVID-19 period, the incidence of bronchiolitis in infants has increased considerably. Several scores determine the degree of severity of the bronchiolitis episode, but few are clinician-friendly. The main aim of this research was to find the easy-to-use score that most accurately estimated the severity of patients’ infections according to their clinical situations and most accurately predicted the need for hospital admission. An observational cross-sectional study was performed in a reference pediatric hospital during the post-restrictions period of the COVID-19 pandemic (2021 and 2022). A comparison was made between the severity estimate provided by five international acute bronchiolitis scales and the clinical severity of the acute bronchiolitis episode. Three hundred and seventy-seven patients participated in the investigation, with a mean age of 5.68 months; 68.7% of the participants had a mild episode of bronchiolitis, 24.5% had a moderate episode, and 6.9% had a severe episode. The severity estimated by the Tal scale modified by McCallum showed a statistically significant correlation with the clinical severity established by clinical criteria (0.836; *p* < 0.001). It showed a high correlation with other international scores, such as the Wang score (0.820; *p* < 0.05) and the Wood–Downes–Ferrés score (0.936; *p* < 0.001). In the multivariate analysis, the constituent variables of the modified Tal score appeared in the final model that predicts the need for hospital admission. In the context of increased incidence after COVID-19, the Tal score modified by McCallum is an easy-to-use measuring instrument that presents an excellent concordance with the clinical severity estimated at first care contact. It also offers a more significant prediction of the need for hospital admission.

## 1. Introduction

Acute bronchiolitis is the first acute respiratory distress episode with pathological auscultation in patients under two years of age. It is also the most common respiratory infection among infants [1]. Its main etiological agents are the respiratory syncytial virus (RSV), followed by the rhinovirus [2]. The highest incidence occurs between November and April, with increased cases in January and February [1,3,4].

This seasonal variation was affected during the COVID-19 pandemic when public health measures caused a considerable decrease in cases of bronchiolitis. With the withdrawal of these measures, there was an increase in infections during atypical times of the year [4,5]. RSV infection accounts for more than 30 million lower respiratory tract infections in children under five years of age, with 3.2 million hospitalizations and 200,000 deaths annually [6].

The clinical diagnosis is based on anamnesis, physical examination, and epidemiology, which does not require complementary tests in most cases [7,8]. Clinical severity can be qualitatively established by correlating various clinical criteria with the location of the patient’s management, whether at home, in the hospital bed, or the intensive care unit [9]. Severity is estimated using validated scores. However, the lack of homogeneity among evaluation systems has hindered the establishment of a reference method [10,11].

Several reviews have evaluated the psychometric properties of the scores. A systematic review and meta-analysis of bronchial obstruction severity scores in pediatric patients analyzed nine of them, concluding, on the one hand, that these tools have excellent interobserver agreement. On the other hand, several weaknesses of these studies could affect their internal validity [12]. Therefore, scientific and medical societies still need to establish the validated scores as a reference system to estimate the severity of acute bronchiolitis, despite previous systematic reviews that evaluated its main psychometric properties [10].

Five such scores were representatively selected from the numerous existing staging instruments. The BROSJOD score was designed in 1999 and validated in 2017 [13]. The parameters of the score are wheezing, muscular retraction, air intake, oxygen saturation (differentiating whether the patient requires oxygen therapy), respiratory rate, and heart rate (the score of these last two variables depends on the patient’s age). Each of the items can be scored from 0 to 3 points. The score provides values from 1 to 16. Three degrees of involvement are established: mild (from 0 to 5 points), moderate (from 6 to 10 points), and severe (from 11 to 16 points).

The ESBA score was designed and validated in 2013 [14]. The parameters of this score are wheezing, crackles, muscle retraction, inspiration–expiration ratio, respiratory rate, and heart rate (the score of these last two variables depends on the patient’s age). Each of the items can be scored score from 0 to 4 points, except for heart and respiratory rates, which can be scored between 0 and 2. The score provides values from 0 to 13. Three degrees of impairment are established: mild (from 0 to 4 points), moderate (from 5 to 9 points), and severe (from 10 to 13 points).

The Tal score modified by McCallum stands out for its simplicity and recent validation. The original Tal score was designed in 1983 [15] and modified by McCallum et al. [16]. This modification was validated by Golan-Tripto et al. in 2018 [17]. The parameters of this score are wheezing/crackles, oxygen saturation, retractions, and respiratory rate (this last item differentiates the score according to age). Each of the items can be scored from 0 to 3 points. The score provides values from 0 to 12. Three degrees of impairment are established: mild (from 0 to 5 points), moderate (from 6 to 8 points), and severe (from 9 to 12 points).

Although its initial use was to rank the severity of asthma, the Wood–Downes score modified by Ferrés is one of the most widely used in clinical practice for evaluating the severity of bronchiolitis. The original score, designed by Wood and Downes in 1972 [18], was modified in 1988 by Ferrés et al. [19]; the modified version is currently most widely used. The parameters of this updated score are wheezing, muscular retraction, cyanosis, respiratory rate, and heart rate. Each of the items can be scored from 0 to 3 points. The score provides values from 0 to 14. Three degrees of impairment are established: mild (from 0 to 3 points), moderate (from 4 to 7 points), and severe (from 8 to 14 points).

The Wang score was designed in 1992 [20]. The parameters of this score are respiratory rate, wheezing, retraction, and general condition. The score provides values from 0 to 12. Three degrees of impairment are established: mild (from 0 to 5 points), moderate (from 6 to 9 points), and severe (from 10 to 12 points).

As mentioned above, none of these scores represents a well-established reference system to stage the severity of patients with acute bronchiolitis.

Given the relevance of hospital admission from a personal and emotional point of view for patients and their families and from the point of view of health system resource management, several systematic reviews in this field consider it of interest to establish an objective measurement tool to estimate the severity of acute bronchiolitis [21]. However, few studies have evaluated scores consisting of simple items that objectively measure respiratory status, are accessible in daily clinical practice, and are readily available at a low cost. To our knowledge, no other research has been performed to assess these aspects in the context of the post-restrictions period of the COVID-19 pandemic.

Therefore, this study aimed to evaluate easy-to-use acute bronchiolitis severity measurement instruments that can more accurately estimate the clinical severity of the patient and the need for hospital admission. We also sought to assess different aspects of these scores in the context of the post-restrictions period of the COVID-19 pandemic and to describe the variables influencing the need for hospital admission.

## 2. Materials and Methods

### 2.1. Study Design

An observational cross-sectional study was performed. Five different bronchiolitis scores estimating the severity of patients 0–24 months of age [1,2] diagnosed with this pathology were compared. The concordance between the severity evaluated by these scores and the clinical situation was assessed.

### 2.2. Study Population

A power calculation of the total number of patients that had to be included was performed. The eligible study population was infants under 24 months of age residing in Almería. According to the National Institute of Statistics, 14,456 children under two years of age were registered in Almeria as of 1 January 2021. The prevalence of bronchiolitis is 20%. Considering a confidence level of 95% (α = 0.05; Zα = 1.96) and a maximum allowed error of 5%, a minimum sample size of 375 infants was calculated.

Discretionary sampling included patients less than 24 months of age with care contacts between October 2021 and February 2022, which is the annual period with the highest incidence of bronchiolitis [1,3,4]. During this period, no COVID-19 restrictions were applied to the general population in Spain. The exclusion criteria were the diagnosis of any other respiratory pathology at discharge, age over 24 months, and refusal to participate in the study.

Upon admission to the Pediatric Emergency Department (PED), a severity level was established based on the clinical status and age of the patient according to other authors [3,4]. Mild bronchiolitis was assessed for patients with an oxygen saturation greater than 92% in ambient oxygen, a respiratory rate less than 60 breaths per minute, adequate oral tolerance, and no apnea in the last 48 h. For these patients, alarm signs were explained, and home management was recommended, with a follow-up with their primary care pediatrician.

Moderate bronchiolitis was assessed for patients who were in a poor general condition, less than 4–6 weeks of age, with an oxygen saturation less than 92% in ambient oxygen with oxygen requirements less than 40%. These patients had a respiratory rate between 60 and 80 breaths per minute (breath/min), partial pressure of CO_2_ greater than 50 mmHg, apnea or cyanosis pauses, respiratory distress, feeding difficulty, presence of signs of pneumothorax, pneumomediastinum without a severe impact on the patient’s general condition, comorbidities, or social risk. They were admitted to the pediatric ward for medium care, if required, for oxygen therapy, fluid therapy, and, in exceptional cases, possible pharmacological treatment.

Severe bronchiolitis was assessed for patients with imminent respiratory failure, a respiratory rate higher than 80 breaths/min or lower than 20 breaths/min with distress, blood pH lower than 7.10 or partial pressure of CO_2_ greater than 60 mmHg, deterioration of the level of consciousness, rapid worsening, acute respiratory distress syndrome, bronchiolitis obliterans, and the need for intubation or mechanical ventilation or oxygen requirements greater than 40%. They were admitted to the pediatric intensive care unit.

The BROSJOD [13], ESBA [14], Tal modified by McCallum [15,16,17], Wood–Downes modified by Ferrés [18,19], and Wang [20] scores were applied, each estimating a mild, moderately mild, or severe severity level depending on the score obtained. In the admitted patients, scores were used at admission and 48 h later to compare the evolution of the patient’s severity during the first days of admission. The score was applied to patients who attended the Pediatric Emergency Department (PED). Scores were applied to patients who attended the PED more than once during the first two visits. The above scores were selected for this study as they consist of simple items that objectively measure respiratory status, are helpful in daily clinical practice, and are readily available at a low cost. All of them stratify patient severity into mild, moderate, and severe.

### 2.3. Data Collection

The medical personnel who attended to the patients in the PED and the admission units collected the necessary clinical data and constants.

The severity of the cases were estimated using the five scores, each presenting three possible values: mild, moderate, or severe. A card was used for each patient to collect examination data and constants. The card included the data and scores to be applied, showing the score and estimated severity.

### 2.4. Statistical Analysis

A descriptive analysis was performed, collecting the frequency and mode of the qualitative variables and the mean, standard deviation, and standard error of the mean of the quantitative variables. The correlation between the estimated clinical severity and initial severity was assessed by each of the scores separately; the concordance between scores 2 to 2 and the correlation between the clinical severity and each of the scores stratified in three age intervals (from 0 to 4 months, from 5 to 10 months, and from 11 to 24 months) [5] were analyzed by applying Goodman and Kruskal’s Gamma coefficient.

The bivariate analysis compared the qualitative and quantitative variables measured between non-admitted patients (mild) and admitted patients (moderate and severe). The Mann–Whitney U test was used for the quantitative variables after testing for normality using the Kolmogorov–Smirnov test, and the Chi-squared test was used for the qualitative variables. A multivariate analysis was performed using a binary logistic regression, calculated with the Intro method, in which the dependent variable was considered income yes/no. The initial independent variables in the model were those that were significant after performing the bivariate analysis.

IBM SPSS Statistics Data Editor version 27.0 software (IBM Inc., Armonk, NY, USA) was used for the data analysis, and a statistical significance level of 95% (*p* < 0.05) was applied.

### 2.5. Ethical and Legal Aspects

This study was conducted according to the standards of good clinical practice and following the international and national standards that regulate Biomedical Research: The Declaration of 3 Helsinki; Law 14/2007 of July on Biomedical Research; to Regulation (E.U.) 2016/679 of the European Parliament and of the Council of 27 April 2016, and Law 3/2018 of December 5 on the Protection of Personal Data and Guarantee of Digital Rights. The research also followed the provisions of Article 12 of Law 14/2007 on Biomedical Research (BOE No. 159 of 4 July 2007). Data for applying the scores were obtained anonymously from the data collected during patient care after obtaining informed consent, and severity was prospectively estimated. This research was approved by the Provincial Research Ethics Committee of Almeria (code: COMPABRON; 5/2022).

## 3. Results

Three hundred and seventy-seven patients, from 1 to 24 months old, participated in the investigation, with a mean age of 5.7 (SD: 5.2) months; 56.5% of the participants were male. A percentage of 68.7% of the participants had mild bronchiolitis, 24.5% had a moderate episode, and 6.9% had severe bronchiolitis.

October and November (41.4% and 31.3%, respectively) were the months with the highest demand for patients with acute bronchiolitis. Statistically significant differences with the remaining months of the study were found (*p* = 0.031).

The mean age of the mild patients was 6.9 months old (SD: 5.2); the mean age of the moderate patients was 3.1 months old (SD: 4.2); and the mean age of the severe patients was 1.9 months old (SD: 2.2). This difference was statistically significant (*p* < 0.001). There were no statistically significant differences in the sex of the patients in the three groups. A total of 68.7% of the patients only required assistance in the PED without admission, and 8% were reconsulted on a second occasion; all were classified as mild cases from a clinical point of view. No statistically significant differences were observed between the mean age of the two groups of the patients who were admitted, while there were differences in the length of the hospital stay (*p* < 0.001).

The correlation between the estimated clinical severity and the severity calculated by the scores is shown in Table 1. The correlation value for the three clinical categories (mild, moderate, and severe) and all age groups depicts the overall assessment of the scores. The Tal score modified by McCallum presents a higher degree of statistically significant correlation (0.836; *p* < 0.001).

The modified Tal score shows a high degree of agreement with the other four compared scores (between 0.733 and 0.959); these four correlations were statistically significant (*p* < 0.05) (Table 2).

In patients younger than ten months old, the Tal score modified by McCallum is the one with the highest degree of correlation with clinical severity in infants younger than four months and aged between 4 and 10 months old (0.855 and 0.974, respectively) (*p* < 0.001). In patients 11 months old or older, the score with the highest concordance with the clinical severity, determined based on the clinical parameters outlined in the Section 2, was the BROSJOD score (0.870; *p* = 0.02) (Table 3).

Table 3 also shows the correlation between severity at admission and after 48 h of the hospital stay, where the Wang score shows a statistically significant correlation (0.781; *p* = 0.002). When the different scores were applied, there were no statistically significant differences between the severity estimated at the first consultation in the PED and the subsequent reconsultation.

Statistically significant differences were observed between patients not admitted (mild) and those admitted (moderate and severe) in mean age, respiratory rate, heart rate, and oxygen saturation values. Significant differences were also observed in the following parameters: altered Pediatric Assessment Triangle, cyanosis, wheezing, crackles, and muscular retraction.

A multivariate analysis was performed using the statistically significant variables between the admitted and non-admitted patients. Table 4 shows the binary logistic regression analysis. The dependent variable considered was hospital admission (admission; yes/no). The initial independent variables in the model were age, sex (men, yes/no), respiratory frequency, heart rate, oxygen saturation, Pediatric Assessment Triangle (stable, yes/no), and presence of wheezing (yes/no), crackles (yes/no), intercostal pull (yes/no), and cyanosis (yes/no).

The final model was composed of the following variables: age, respiratory frequency, oxygen saturation, presence of wheezing, and presence of muscular retraction. In admitted patients, the lower the age, the higher the respiratory frequency, the lower the oxygen saturation, the lower the presence of wheezing, and the greater the presence of respiratory distress (OR: 0.7454, OR: 0.623, OR: 0.605, and OR: 2.223, respectively).

## 4. Discussion

The present study analyzes and compares different easy-to-use scores used to objectively establish the severity of acute bronchiolitis in infants to determine which score best predicts the need for hospital admission and correlates best with the clinical situation. This is the first research to assess these aspects in the context of the post-restrictions period of the COVID-19 pandemic.

The sample size of our research was more significant than that of others used in studies on similar subjects, which generally ranged between 30 and 115 patients [12,22,23,24], and which was slightly surpassed by the study of Marlais et al. [25]. The age of our patients was similar to that of those who participated in other studies. The study was conducted in 2021 and 2022 during the post-restrictions period of the COVID-19 pandemic, making this study unique.

In patients with moderate or severe bronchiolitis, those requiring oxygen therapy, or other hospital care, it is essential to establish the correct criteria for hospital admission due to the possible emotional repercussions on the patient’s family [25,26,27]. In a prospective multicenter study of 117 patients from 17 centers in the 2018 and 2019 bronchiolitis season, 40% of families reported a negative impact on family quality of life during the first week of their stay at the clinic [28]. A 2019 systematic review of parents’ experiences during their children’s hospital stays to treat acute bronchiolitis stated that hospitalization was a stressful and distressing situation for them, in which mothers felt that they did not have an active role in caring for their children [27].

In addition to emotional implications, there are financial implications of admission for bronchiolitis. Treatment can cost approximately 1300 USD per hospitalized patient [28]. Nearly 40% of the parents of children under eight weeks of age in New York State requested sick leave to care for their children with lower respiratory tract infections [29]. Due to these economic consequences for the health care system and the emotional implications for family members and patients, it is essential to find the best method to objectively determine the severity of this pathology and the need for hospital admission.

Numerous studies and analyses have been conducted on existing bronchiolitis severity scores without scientific and medical societies establishing them as the reference system for measuring bronchiolitis severity [21]. The scores compared in a systematic review and meta-analysis showed good interobserver agreement [12]. Another systematic review published in 2018 stated that few instruments have been validated to estimate the severity of bronchiolitis. The authors also noted that identifying an optimal measurement instrument to assess the severity of bronchiolitis will contribute to good clinical practice and research [21].

Instruments to estimate the severity of acute bronchiolitis are highly variable in composition, and some of them are adaptations or modifications of other existing scores [18]. A score modified after its creation and analyzed in the present study is the Tal score, modified by McCallum in 2013 [15,16] and validated by Golan-Tripton in 2018 [17]. Two studies on patients admitted for acute bronchiolitis agree that the modified Tal score has high reliability [15,23] and constitutes a repeatable system [16]. Another study concludes that this score has adequate predictive validity [22]. The repeatability and reliability observed in these previous studies are consistent with the results of our research. In our case, the data are collected by numerous professionals, presenting a high level of precision and a coincidence between the severity calculated with Tal’s score and that estimated by the patient’s clinical situation.

Whereas the need for hospital admission is determined by clinical severity, our study agrees that the modified Tal score has adequate predictive validity as the score that may best predict the final variable of the need for hospital admission. This finding reinforces the fact that the Tal score modified by McCallum is the one that presents the highest degree of agreement between the estimated severity and the clinical severity of the patients, since this relationship is statistically significant.

According to our results, the Tal score shows more significant agreement with clinical severity; however, it is necessary to analyze the literature and compare it with other instruments. One study compared the Tal score with the Wood–Downes–Ferrés score and concluded that there was a statistically significant positive correlation between them. The study also described that the Tal score had sufficient content validity, adequate convergence criterion validity, and adequate sensitivity to change after this comparison [12]. The conclusions of this study coincide with ours in that there is also a very high statistically significant correlation between the Tal score and the Wood–Downes–Ferrés score (0.936; *p* < 0.001).

Another study conducted on admitted patients concluded that the modified Tal score presents greater validity, reliability, sensitivity, and specificity than the Wang score in estimating the severity of respiratory diseases in children [24]. Our study found that the Wang score [20], designed in 1992, is in concordance with the estimated clinical severity (0.783; *p* < 000.1), although lower than that provided by the modified Tal score, which is a finding that coincides with the findings of the Jakarta study. The degree of concordance between both scores in our study is high. This relationship is statistically significant (0.820; *p* < 0.001). Therefore, the modified Tal score seems slightly superior in concordance with the estimated clinical severity compared to other international scores.

Concerning the comparison between scores, a bibliographic review states that Lowell’s RDAI score is the most widely used in daily clinical practice according to the literature consulted, followed by the modified Tal and Wang scores. These last two scores have been included in our study; however, we excluded the RDAI score from our analysis, since it only establishes two categories of severity (mild-moderate and severe), where the limit is 8 points [30]. The RDAI score has been subjected to the most rigorous psychometric tests, but its construct validity is moderate, and its superiority over the other compared scores has not been demonstrated [10]. Our results show that although different scores may correlate, they do not need to correlate equally with specific clinical parameters that allow a quick and easy clinical evaluation of patients with bronchiolitis. Therefore, one of the significant contributions of this study is to identify which of these scales, which are simple and quick to use, correlates best with clinical parameters that provide simple and quick evaluation methods [3].

By comparing the different scores, the present study highlights the relevance of the Tal score over other scores. There are previous studies in this field, although smaller than the one we have performed. One of them, conducted in 2016 on 201 patients, compared the ESBA score with the Wood–Downes–Ferrés score. It stated that none of the scores analyzed were considered ideal for evaluating their patients, making it necessary to perform further studies to optimize the severity estimation using these scores [31]. In our study, both scores show moderate agreement with each other. The concordance with Tal’s score is high or very high, respectively.

After analyzing the comparative literature on the different scores, only a few studies that identify the score that best predicts the need for hospital admission were found; these studies are in agreement with our study on the superiority of the modified Tal score. Few studies have attempted to establish clear admission criteria using scores. A retrospective descriptive study of 449 infants with acute bronchiolitis seen in a PED identified the duration of symptoms, respiratory rate, heart rate, oxygen saturation, and age at presentation as clinical predictors of hospital admission [25]. The Marlais score was composed of these five predictors; they were scored from 0 to 1, with a maximum score of 5 points. Our study obtained a final prediction model that coincides with the Marlais score on three of the five criteria (respiratory frequency, oxygen saturation, and age at presentation) and all of these predictors were variables used in the Tal score modified by McCallum. Regarding wheezing, the presence of this sign was a protective factor since the absence of wheezing may be due to hypoventilation, implying a more severe clinical picture. However, the agreement with the Tal score modified by McCallum is much higher since the model reflects most of the parameters of this scale.

This study has some limitations. One of the most important limitations is selection bias; participating individuals were included based on discretionary sampling in a single center. This aspect opens the opportunity to conduct similar studies in populations with other demographic and epidemiological characteristics. Another limitation is the comparison of a limited number of scores. Numerous staging instruments prevent comparing all of them in a single study, which is conducive to developing other projects. Finally, none of the analyzed scales shows the highest concordance with the clinic in all the circumstances studied, such as in older infants or patients admitted after 48 h of stay.

This study also has some strengths: the most important strength was using a sample size of 377 patients, which is considerably higher than other studies conducting similar research. Another strength to highlight is the inclusion of five easy-to-use national and international scores for comparison with each other and the patient’s clinical situation, which is something new in comparative studies according to the current literature. This comparison provides helpful information in clinical practice to optimize resources and avoid unnecessary admissions for this pathology. An additional strength is the analyzed bronchiolitis season. This is one of the few comparative studies of bronchiolitis scores during the post-restrictions period of the COVID-19 pandemic. As a final strength, the five scores compared in our study are made up of simple items of objective and practical measurement without requiring a high cost or a significant learning curve for professionals, which facilitates carrying out the study effectively and efficiently.

## 5. Conclusions

This research is the first to assess easy-to-use severity scores of acute bronchiolitis in the post-restrictions period of the COVID-19 pandemic. The Tal score modified by McCallum is the measurement instrument with the highest concordance with the estimated clinical severity at first care contact, and the highest predictor of the need for hospital admission. The modified Tal score shows a high degree of concordance with other instruments for estimating the severity of acute bronchiolitis, such as the Wang and Wood–Downes–Ferrés scores.

## Figures and Tables

**Table 1:** Correlation between clinical severity and severity estimated by each score.

	Correlation Value	*p*-Value ^1^
BROSJOD	0.711	<0.001
ESBA	0.653	<0.001
Tal modified by McCallum	0.836	<0.001
Wood–Downes–Ferrés	0.467	<0.001
Wang	0.783	<0.001

^1^ Goodman and Kruskal’s Gamma coefficient.

**Table 2:** Correlation between severity estimated by Tal score vs other scores.

	Correlation Value	*p*-Value ^1^
BROSJOD	0.959	<0.001
ESBA	0.733	<0.001
Wood–Downes–Ferrés	0.936	<0.001
Wang	0.820	0.04

^1^ Goodman and Kruskal’s Gamma coefficient.

**Table 3:** Score with the highest degree of correlation with clinical severity according to age (in intervals) and with the evolution of patients who return to the PED and those admitted 48 h after admission.

			Correlation Value	*p*-Value *
Clinical severity	BROSJOD	0 to 4 months old	0.770	<0.001
		5 to 10 months old	0.908	<0.001
		11 to 24 months old	0.870	0.021
	ESBA	0 to 4 months old	0.789	<0.001
		5 to 10 months old	0.634	0.011
		11 to 24 months old	0.265	0.590
	Tal modified by McCallum	0 to 4 months old	0.855	<0.001
		5 to 10 months old	0.974	<0.001
		11 to 24 months old	0.800	0.107
	Wood−Downes−Ferrés	0 to 4 months old	0.463	<0.001
		5 to 10 months old	0.698	0.003
		11 to 24 months old	0.856	0.022
	Wang	0 to 4 months old	0.813	0.001
		5 to 10 months old	0.724	0.002
		11 to 24 months old	0.779	0.029
Ped reconsulting	BROSJOD		0.000	1.000
	ESBA		−0.048	0.335
	Tal modified by McCallum		**	
	Wood−Downes−Ferrés		0.449	0.148
	Wang		−0.289	0.574
48 h after admission	BROSJOD		0.762	0.028
	ESBA		−0.441	0.154
	Tal modified by McCallum		0.000	1.000
	Wood−Downes−Ferrés		0.313	0.102
	Wang		0.781	0.002

* Goodman and Kruskal’s Gamma coefficient. ** Statistics have not been calculated because “Tal PED reconsulting” is a constant.

**Table 4:** Multivariate analysis: binary logistic regression of the variables influencing the need for hospital admission (dependent variable: admission yes/no).

Variable	aOR	CI	*p*-Value
Age	0.754	0.677–0.841	<0.001
Respiratory rate	1.052	1.017–1.089	0.003
Oxygen saturation	0.623	0.536–0.724	<0.001
Wheezing	0.605	0.396–0.924	0.02
Respiratory distress	2.223	1.559–3.172	<0.001
Altered air entry	0.692	0.451–1.061	0.091

aOR: adjusted Odds Ratio; CI: 95% Confidence Interval.

## Data Availability

The data presented in this study are available on request from the corresponding author. The data are not publicly available due to privacy and ethical restrictions.
